# Availability of an Assay for Detecting *Mycobacterium tuberculosis*, Including Rifampin-Resistant Strains, and Considerations for Its Use — United States, 2013

**Published:** 2013-10-18

**Authors:** Div of Tuberculosis Elimination, National Center for HIV/AIDS, Viral Hepatitis, STD, and TB Prevention, CDC

In August 2013, the Food and Drug Administration (FDA) permitted marketing of the Xpert MTB/RIF assay (Cepheid, Sunnyvale, California) to detect DNA of the *Mycobacterium tuberculosis* complex (MTBC) and genetic mutations associated with resistance to rifampin (RMP) in unprocessed sputum and concentrated sputum sediments (1). Along with clinical, radiographic, and other laboratory findings, results of the assay aid in the diagnosis of pulmonary tuberculosis (TB). The assay is a nucleic acid amplification-based (NAA)* test using a disposable cartridge in conjunction with the GeneXpert Instrument System. Sensitivity and specificity of the Xpert MTB/RIF assay for detection of MTBC appear to be comparable with other FDA-approved NAA assays for this use, although direct comparison studies have not been performed. Sensitivity of detection of RMP resistance was 95% and specificity 99% in a multicenter study using archived and prospective specimens from subjects aged ≥18 years suspected of having TB who had 0–3 days of antituberculous treatment (1). CDC continues to recommend following published U.S. guidelines for TB diagnosis and infection control practice, including the use and interpretation of NAA test results (2–4). Providers and laboratories need to ensure that specimens are available for other recommended mycobacteriological testing. The Xpert MTB/RIF assay aids in the prompt diagnosis of TB and RMP-resistant disease. RMP resistance most often coexists with isoniazid (INH) resistance; TB that is resistant to both drugs is multidrug-resistant (MDR)† TB. Because the prevalence of RMP resistance is low in the United States (about 1.8% of TB cases) (5), a positive result indicating a mutation in the *rpoB* gene of MTBC should be confirmed by rapid DNA sequencing for prompt reassessment of the treatment regimen and followed by growth-based drug susceptibility testing (DST) (1,6,7). CDC offers these services free of charge.§

The World Health Organization has published guidance on use of the Xpert MTB/RIF assay aimed primarily at settings where the prevalence of TB and drug-resistant disease is much higher than in the United States (8).

## Detection of MTBC

In 2008, the Association of Public Health Laboratories and CDC convened a panel¶ that recommended NAA testing as standard practice in the United States to aid in the initial diagnosis of patients with suspected TB. On the basis of the panel report (7) and consultation with the Advisory Council for the Elimination of TB, CDC published revised NAA guidelines, including a detailed testing and interpretation algorithm for initial diagnosis (4). Recent studies further support NAA test use in the United States to avoid delays in diagnosis and treatment, especially for patients with suspected TB and sputum smears negative for acid-fast bacilli on microscopy. Because of rapid results, NAA testing can help avoid unnecessary respiratory isolation, treatment, and contact investigation of patients without TB (9)** and can contribute to system cost savings in patients with HIV infection, homelessness, or substance abuse, compared with smear microscopy alone (9).

CDC recommends that NAA testing be performed on at least one (preferably the first) respiratory specimen from each patient suspected of pulmonary TB for whom a diagnosis of TB is being considered but has not yet been established, and for whom the test result would alter case management or TB control activities (4). The recommendation emphasizes the need for NAA testing in the initial diagnosis and for triaging public health interventions such as contact investigations and infection control decisions. Parallel guidance for the use of NAA TB testing in patients infected with HIV has been published (10). NAA testing does not replace the need for culture; all patients suspected of TB should have specimens collected for mycobacterial culture (4).

## Practical Considerations for Use of the Xpert MTB/RIF Assay for Detection of Mutations Associated with RMP Resistance

CDC presents these interim practical considerations for incorporation of the Xpert MTB/RIF assay into diagnostic algorithms. For any test, even with high sensitivity and specificity (including NAA testing, DNA sequencing, and growth-based DST), the positive predictive value is low for a rare condition; accordingly, health-care providers should consult with their public health laboratory for confirmation by rapid molecular detection of mutations associated with drug resistance. To confirm a positive result, genetic loci associated with RMP resistance (to include *rpoB*), as well as INH resistance (to include *inhA* and *katG)* should be sequenced to assess for MDR TB. If mutations associated with RMP resistance are confirmed, rapid molecular testing for other known mutations associated with drug resistance (to first-line and second-line drugs) is needed for health-care providers to select an optimally effective treatment regimen. All molecular testing should prompt growth-based DST (6,7). Laboratories should report an Xpert MTB/RIF assay positive for RMP resistance pending confirmatory results (Table 1). Note that although an Xpert MTB/RIF assay result positive for MTBC and negative for RMP resistance has high negative predictive value†† for ruling out RMP resistance, growth-based DST to first-line TB drugs is still necessary. Consultation with a TB expert is recommended if the clinician is not experienced in the interpretation of NAA and other molecular test results or the diagnosis and treatment of TB. This is especially important in cases of suspected drug resistance (3).

## Considerations for Infection Control

CDC recommends airborne infection isolation (AII) precautions for patients with suspected TB disease of the lungs, airway, or larynx in health-care settings (3,10). AII precautions may be discontinued when contagious TB disease is considered unlikely and either 1) another diagnosis is made that explains the clinical syndrome or 2) the patient has three consecutive sputum smears negative for acid-fast bacilli on microscopy. Because of the intermittent presence of TB bacilli in the sputum of patients with TB, three specimens separated in time have been recommended to have a sufficiently high predictive value for excluding contagious disease.

Because NAA testing, including that with the Xpert MTB/RIF assay, is significantly more sensitive and specific for the detection of MTBC than microscopy alone, substitution of Xpert MTB/RIF assay results that are negative for MTBC for microscopy results increases the negative predictive value for MTBC. Therefore, in ruling out contagious TB, specimens can be tested by microscopy, NAA, or a combination of the two (Table 2) (2,10). Three sputum specimens, each collected 8–24 hours apart, with one being an early morning specimen, should be collected to inform decisions regarding the discontinuation of AII precautions for patients with suspected TB in health-care settings. For patients with a diagnosis of TB, decisions regarding discontinuation of AII precautions should be based on microscopy (i.e., three consecutive negative smears) and other clinical criteria (10).

## Figures and Tables

**TABLE 1:** Interpretation and proposed minimum laboratory report laboratoy for results from the Cepheid Xpert MTB/RIF assay* — United States 2013

GeneXpert Instrument System generated result using Xpert MTB/RIF assay	Interpretation of Xpert MTB/RIF assay result	Minimum laboratory report language†
MTB detected, RIF resistance detected	MTB target is detected within the sample. A mutation§ in the *rpoB* gene has been detected.	MTBC detected. A mutation in *rpoB* gene has been detected, indicating possible RMP resistance. Confirmatory testing should follow.¶
MTB detected, RIF resistance not detected	MTB target is detected within the sample. A mutation in the *rpoB* gene has not been detected.	MTBC detected. No *rpoB* gene mutations detected; probably RMP susceptible.
MTB detected, RIF resistance indeterminate	MTB target is detected within the sample. A mutation in the *rpoB* gene because of insufficient signal detection.	MTBC detected; presence of *rpoB* gene mutations cannot be accurately determined.
MTB not detected	MTB target is not detected within the sample.	MTBC not detected.

**Abbreviations:** To be consistent with the Xpert MTB/RIF assay package insert, MTB and MTBC = *Mycobacterium tuberculosis* complex; and RIF and RMP = rifampin.

* All samples tested by the Xpert MTB/RIF assay should have concomitant mycobacterial culture, regardless of the Xpert MTB/RIF assay results, to address lower sensitivity of the Xpert MTB/RIF for sputum samples that are negative on acid-fast bacilli microscopy, and to obtain isolates for drug susceptibility testing and genotyping.

† CDC suggested minimum language for the laboratory report. Laboratories are encouraged to enhance and customize this basic language in accordance with the capabilities or referral systems of their institution.

§ Might refer to more than one mutation.

¶ Because of the low positive predictive value of RMP resistance results in low prevalence populations, in the United States, confirmatory testing should include prompt DNA sequencing and subsequent phenotypic drug susceptibility testing of cultured isolates. DNA sequencing of direct patient samples (or if not available, isolates) with possible RMP resistance should include genetic loci associated with resistance to RMP (to include *rpoB*) as well as isoniazid (to include *inhA* and *katG*) to assess for multidrug-resistant tuberculosis; *rpoB* mutations detected by the Xpert MTB/RIF assay might be silent mutations that do not affect RMP susceptibility. DNA sequencing can distinguish silent mutations, which in this context refer to synonymous single nucleotide polymorphisms (also known as sSNPs).

**TABLE 2:** Analysis of results of SS and NAA testing for infection control in health-care settings involving patients with suspected tuberculosis (TB) — United States, 2013

Results of combinations of SS and NAA testing on a total of at least three specimens, each collected 8–24 hours apart, with one being an early morning specimen		
	
SS results	NAA test results	Decisional analysis
All SS tests are negative.	All NAA tests are negative for the detection of MTBC.	In combination with other requirements,* supports discontinuation of airborne infection isolation (AII) precautions.
At least one SS test is positive.	All NAA tests are negative for the detection of MTBC.	Consistent with presence of NTM; pulmonary or laryngeal TB is unlikely but cannot be excluded, pending culture results and other clinical determinants. Decision to discontinue AII precautions based on evaluation of all clinical information and potential risk for transmission.
At least one SS is positive.	At least one NAA test is positive for the detection of MTBC.	Consistent with pulmonary or laryngeal TB; supports continuation of AII precautions until recommended criteria are met.*
At least one SS is positive.	At least one Xpert MTB/RIF assay is positive for the detection of MTBC and rifampin (RMP) resistance.	Consistent with suspected pulmonary or laryngeal MDR TB; Xpert MTB/RIF assay rifampin resistance result should be confirmed by rapid DNA sequencing and be accompanied by first and second line growth-based DST; if RMP resistance is confirmed, some infection-control practitioners may choose AII precautions during the entire hospitalization or until culture conversion is documented.*
SS is not available.	At least one NAA test is positive for the detection of MTBC.	Consistent with pulmonary or laryngeal TB; SS needed to contribute to infection control decision making.
All SS are negative.	At least one NAA test is positive for the detection of MTBC.	Consistent with pulmonary TB but less contagious where three SS are negative; may support discontinuation of AII precautions if other criteria for discontinuing AII precautions are met (e.g., patient has received a sufficient duration of effective TB treatment) or patient is housed in setting where risk for transmission is low and treatment is started promptly.
All SS are negative.	At least one Xpert MTB/RIF assay is positive for the detection of MTBC and RMP resistance	Consistent with suspected pulmonary TB but less contagious where three SS are negative; the Xpert MTB/RIF assay rifampin resistance result should be confirmed by rapid DNA sequencing and be accompanied by first and second line growth-based DST; if confirmed as RMP resistant, some infection-control practitioners may choose AII precautions during the entire hospitalization, or until culture conversion is documented.*

**Abbreviations:** To be consistent with the Xpert MTB/RIF assay package insert, MTB and MTBC = *Mycobacterium tuberculosis* complex; and RIF and RMP = rifampin. SS = sputum-smear microscopic examination for acid-fast bacilli; NAA = nucleic acid amplification; MTBC = *Mycobacterium tuberculosis* complex; AII = airborne infection isolation; NTM = nontuberculous mycobacteria; MDR = multidrug-resistant (defined as resistance to at least isoniazid and RMP); DST = drug susceptibility testing.

* **Source:** CDC. Guidelines for preventing the transmission of Mycobacterium tuberculosis in health-care settings, 2005. MMWR 2005;54(No. RR-17).

## Footnotes

* “NAA tests” are only one type of “direct” molecular diagnostic devices that are applied to a clinical specimen (e.g., sputum) with or without processing. NAA testing to diagnose MTBC in patients should not be confused with testing using NAA assays applied to isolates for the rapid species identification of MTBC.

† MDR TB is defined as TB caused by MTBC resistant to at least INH and RMP. Extensively drug-resistant TB (XDR TB) is a type of MDR TB that is additionally resistant to any fluoroquinolone and to at least one of three injectable second-line drugs (i.e., amikacin, kanamycin, or capreomycin).

§ Information available at http://www.cdc.gov/tb/topic/laboratory.

¶ In response to a recommendation by the Advisory Council for the Elimination of Tuberculosis.

** Davis JL, Ho C, Cattamanchi A, et al. The clinical and public health impact of automated nucleic acid testing for TB evaluation in San Francisco. Abstract. American Thoracic Society International Conference. Denver, Colorado; 2011. Available at http://www.atsjournals.org/doi/pdf/10.1164/ajrccm-conference.2011.183.1_meetingabstracts.A5314.

†† Approximately 5% of RMP resistance is not associated with *rpoB* mutations. Combined with a RMP resistance prevalence of about 1.8% in United States, the negative predictive value is high for RMP resistance when *rpoB* mutations are not found. Because culture-based DST is recommended for all cases of TB, almost all RMP resistance can be detected.
